# Resilience-based optimization model for emergency bus bridging and dispatching in response to metro operational disruptions

**DOI:** 10.1371/journal.pone.0277577

**Published:** 2023-03-29

**Authors:** Jiefei Zhang, Gang Ren, Jianhua Song

**Affiliations:** 1 School of Energy and Safety, Anhui University of Science and Technology, Huainan, Anhui, China; 2 Jiangsu Key Laboratory of Urban ITS, Southeast University, Nanjing, China; 3 Jiangsu Province Collaborative Innovation Center of Modern Urban Traffic Technologies, Southeast University, Nanjing, China; 4 School of Transportation, Southeast University, Nanjing, Jiangsu, China; Southwest Jiaotong University, CHINA

## Abstract

Bus-bridging evacuation services can significantly enhance metro resilience during operational disruptions. A resilience-based optimization model was proposed to generate a bus bridging and dispatching plan. The objective of the model is to maximize the resilience index of evacuated passengers while meeting pre-established restrictions on operational indicators and resources. The proposed approach consists of three steps: representing an integrated network based on a hyper-network, generating candidate bus-bridging routes using the K-shortest paths algorithm, and solving the optimization model using a genetic algorithm to determine the optimal vehicle allocation among the candidate routes. The Nanjing metro network was used to demonstrate the proposed model. The results show that the average waiting time is the main reason for travel delays, especially in short-distance travel. Furthermore, the cycling strategy is beneficial for reducing the average travel delay and improving evacuation efficiency with limited vehicles. In particular, when resources are very limited, the vehicle cycling strategy may have significant advantages over fixed vehicles for servicing fixed lines. The proposed model could be widely used in emergency response to quickly and efficiently evacuate passengers.

## 1. Introduction

As one of the rapid and high-capacity means of transportation, metro systems have become an effective way of addressing congestion problems in big cities. However, the system is often vulnerable to natural disasters, terrorist attacks, and equipment failures. Partial lines or sections may be inoperable owing to those disruptions. In such scenarios, numerous passengers must be evacuated in a timely and expertly manner. During metro disruptions, buses dispatched from depots or retracted from existing bus routes can operate in parallel with the affected metro line. The design of temporary bus services and routes to restore connectivity between the undisrupted metro lines can be called “bus bridging.” These temporary bus services can “bridge” impacted metro stations and efficiently evacuate the affected passengers. Thus, in practice, most metro operating companies formulate particular bus bridging emergency plans to alleviate passenger delays and guarantee metro reliability.

More attention is paid to bus bridging design and optimization after the metro system is disrupted. The methodological framework for bus bridging, which was first proposed by Kepaptsoglou and Karlaftis [1], can be divided into three parts: (a) definition of the bridging environment, (b) design of bridging routes, and (c) allocation of resources. Jin et al. [2] suggested three steps to optimize bus bridging services: (a) generating demand-responsive candidate bus routes, (b) identifying the most effective combination of candidate bus routes, and (c) determining the optimal allocation of available vehicles among the selected routes. Some studies have followed an integrated framework consisting of three parts: network design, line planning, and bus resource allocation. Only a few studies have focused on one or two of these steps. For example, Gu et al. [3] used predefined bridging routes as input and only optimized the allocation of the bus resources. Deng et al. [4] designed the bus bridging routes in response to disruption of metro. The proposed model did not consider the bus resource allocation and only focused on the route design.

It is generally assumed that, similar to regular transit services, bridging buses operate exclusively on predetermined routes with fixed frequencies. For example, Wang et al. [5] proposed a model for optimizing the path for each bus to minimize total bus travel times. Cadarso et al. [6, 7] developed an integrated optimization model for bus bridging timetable and rolling stock rescheduling to minimize recovery time, passenger inconvenience, and incurred system costs. Wu et al. [8] considered passenger delay in the bus bridging and metro short-turning processes to develop a coordinated emergency response model for metro disruptions. Li et al. [9] formulated an integrated optimization model to address both bus bridging route layouts and bus resource allocation. The stop-by-stop, express, and skip-stop route schemes, parallel to the disrupted line segment, were applied in the optimized bridging scheme to best respond to passenger demands. Luo and Xu [10] developed a stochastic programming model for the design of bus bridging services plan within bus resource limit. The objective of the model is to minimize the expected unsatisfied commuter demand, considering uncertainties in commuter demand and spare capacities of existing rail and bus lines. Liang et al. [11] constructed a robust bus bridging service design. The robust model was formulated to incorporate bus travel time uncertainty. The model focused on minimizing total passenger and operational costs by optimizing passenger flow and bus bridging line frequency. The above research assumes the bridging bus operates the route with a fixed frequency.

However, fixed frequencies may be suitable for expected or long-term disruptions. In contrast, during unexpected and short-term disruptions, thousands of metro passengers are left stranded at the affected stations. Given the high demand and limited bus resources, the bus bridging strategy should be changed to evacuate stranded passengers as soon as possible. In this case, the bus fleet should be quickly dispatched to the affected stations and require no fixed frequency or headway. This strategy improves service flexibility and operational efficiency for unexpected and short-term disruptions. Gu et al. [3] proposed a flexible bus bridging strategy in which each bus operates based on a bridging plan that sequentially lists the stations to serve, instead of route frequencies. A two-stage model was developed to optimize the bridging plans aiming to minimize bus bridging time and passenger delay. A heuristic algorithm based on the weight shortest processing time (WSPT) rule was used to solve the proposed model. Hu et al. [12] presented a nonlinear integer programming model for better planning bus bridging evacuation during metro disruptions. The objective of the model is to minimize the maximum evacuation time. A customized genetic algorithm (GA) with a specially designed mutation mechanism was designed to efficiently solve the model. Hu et al. [13] presented an integrated planning and operational model for bus bridging evacuation, without considering frequency. The bus fleet operates along an optimally planned route to minimize the total operational cost. The implemented dispatch optimization procedure was based on a GA. Wang et al. [14] established a bi-level programming model for bus bridging optimization. The upper-level model was a timetable optimization for minimizing passenger transfer costs. The lower-level model was a vehicle-scheduling plan for minimizing bus operation costs. The study also compared the results of the even- and uneven-headway timetables and found that the uneven-headway plan can reduce the total system and waiting time costs, although increasing the operational costs. Chen et al. [15] adopted a strategy in which buses could flexibly serve different bridging routes without frequency. They proposed a bus emergency centralized cycle dispatching optimization model to minimize the time required for the total evacuation of all stranded passengers.

This paper focuses on the evacuation of passengers suddenly left stranded from metro stations affected by short-term disruptions to unaffected stations. To this aim, a flexible cycle dispatching strategy is adopted to reduce no-load travel and improve evacuation efficiency. Specifically, vehicle cycling refers to allowing one vehicle to serve a completely different route in another direction after it has finished its allotted route. In other words, vehicle cycling means that some vehicle trajectories will be circular. When the vehicle finishes servicing the allotted route, it needs to immediately serve a route in another direction. Thus, the model in this study ignores bus frequency but considers vehicle turn-back time.

In existing research, bus bridging optimization objectives can be divided into two categories. One is minimizing the evacuation time of bridging-buses [15–18] or operating cost [13, 20] from the perspective of operation management. The other is minimizing the travel delay [19], total waiting time [14, 20]and un-evacuated demand [10, 20–22] from the perspective of passenger. When dealing with multi-objective optimization, some studies have adopted multi-stage optimization model, where each stage has a different objective [10, 14, 19, 23]. Other studies have adopted an integrated optimization model whose optimization objective includes both the operational cost and stranded passenger delay [8, 9, 21]. The algorithm of the model can be classified into two broad categories: analytical and heuristic [1]. The former suits for small scale scenario [10]. The latter can be adopted to large scale scenario. For example, genetic algorithm [12, 13, 16, 20], tabu search [21], simulated annealing algorithm [14] are used in the previous research.

However, these optimization objectives and procedures always ignore the evacuation process and focus only on the final result. In general, evacuation often lasts many hours; therefore, the evacuation process should be emphasized. To fill this gap, this study adopts a resilience analysis to evaluate the changed state. Infrastructure resilience is defined as its ability to absorb, adapt to, and rapidly recover from potentially disruptive or destructive events. A resilient system can better reduce the risk of inevitable disruptions or accidents and ensures that the system functions elegantly degrade [24]. All kinds of research have been conducted to evaluate and quantify system resilience, as it can better reflect the change in system performance over time [25–27]. The resilience of the system can be quantified by a resilience index, which is equal to the ratio of the areas covered by the recovery and normal performance curves during a set period [25, 26]. This study adopts the resilience index to quantify the number of evacuated passengers.

A few research was done that combined resilience evaluation and bus bridging optimization. Jin et al. [22] proposed a two-stage stochastic programming model to assess the metro network resilience as well as to optimize the localized integration with bus services. The first stage is to maximize the expectation of the fraction of satisfied travel demand. The second stage is to maximize demand fulfillment. But the model only considered the final result. De-Los-Santos et al. [28] measured robustness index relative to the overall travel time of a metro network. They also compared the robustness index without-bridging interruptions and with-bridging interruptions. But their work only gives insights into the robust evaluation in the bus bridging scenario. Aboudina et al. [29] presented a practical tool to evaluate the total users’ delay associated with a user-specified bus bridging plan based on deterministic queueing theory. But this research only focuses on bus bridging evaluation.

Combining resilience evaluation with bus bridging and dispatching optimization is meaningful and innovative for emergency evacuation after metro operational disruptions. This can make the evacuation more efficient and minimize the delay of affected passengers from the view of the entire evacuation process. This study enriches the research on bus bridging optimization based on resilience.

Focusing on short-term and unexpected metro disruptions, this paper proposes an emergency bus bridging and dispatching optimization procedure to maximize the resilience index of evacuated passengers while meeting pre-established thresholds for the average travel delay and vehicle resources. The proposed procedure comprises an integrated network based on a hyper-network, generates candidate bus bridging routes using the K-shortest paths algorithm, adopts a cycling operation strategy, and proposes an optimization model based on resilience to determine the optimal allocation of the available vehicles among the candidate routes. A GA is adopted to solve the dispatching model. In addition, the vehicle cycling strategy can fully use the bus capacity and reduce the no-load travel taking into account the entire evacuation process. The proposed procedure and model can guide practical evacuation tasks to enhance public transit resilience and improve emergency response efficiency during short-term and unexpected metro disruptions.

The rest of this paper is organized as follows. Section 2 proposes a novel resilience-based model to optimize the bus-bridging and dispatch from the view of the entire evacuation process. In section 3, the Nanjing metro is used to illustrate the proposed model. Conclusion and future work are concluded in section 4.

## 2. Methodology

### 2.1 Problem assumptions

**Fig 1:**
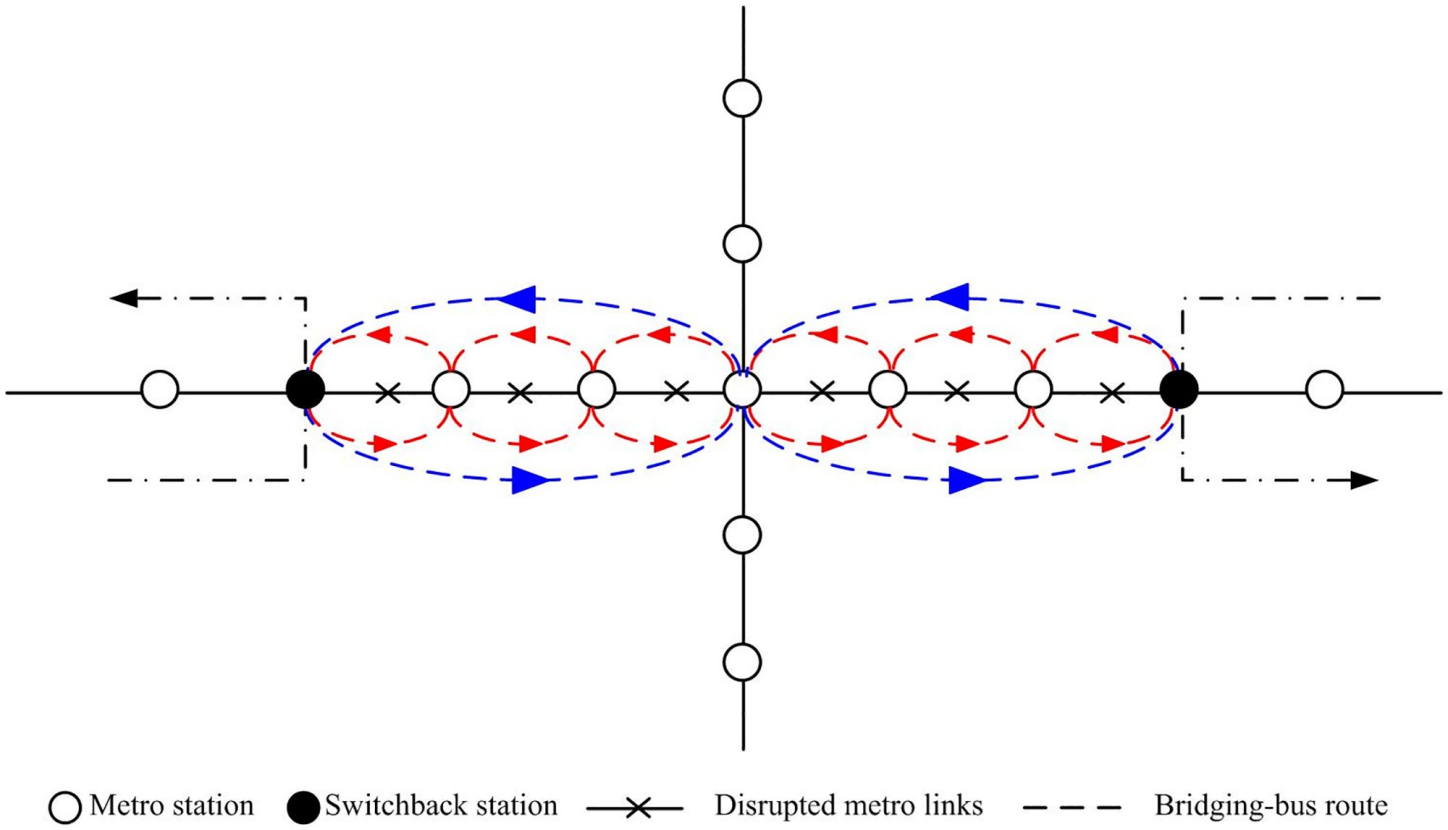
Schematic of metro network disruption and bus bridging response routes.

If an operational disruption lasts more than 30 min, it is necessary to start bus bridging services. In practice, many turn-back stations exist in the network, and trains can return to those stations that are closest to the interrupted links. Thus, the disruption affects the links between turn-back stations, as shown in Fig 1. In such a situation, the standard response is to run bridging buses parallel to the disrupted metro links, along “standard bridging routes,” such as the ones indicated by red lines in Fig 1. However, this method is not often the optimal response because it ignores the pattern of passenger travel demand and may cause larger delays. Instead, the response can be improved by running other vehicles along additional routes, such as those shown by the blue lines in Fig 1. The combination of these routes can decrease the stop time and average delay for the passengers. Because the capacity of the buses is smaller than that of the metro, a multicycle bus dispatch strategy needs to be adopted. In contrast to previous studies, where each vehicle only serves a specific origin-destination (OD) demand [2, 6], in this study vehicles are allowed to run on different routes in different directions.

To simplify the complexity of the problem, the following assumptions are made:

1. The affected passenger travel demand is known and can be represented by an OD matrix. After the disruption, when passengers are informed of the metro disruption, they will give up the metro system and choose other modes of transportation to finish their trip. Thus, the total evacuation demand includes both the passengers stranded at the start of the disruption and those arriving shortly after. This information can be easily estimated from historical data owing to the implementation of automated fare collection systems [3]. However, this study ignores the influence of alternative means of transportation, such as cars and taxis.
2. The capacity, speed, and boarding time are the same for all buses, and their riding time is proportional to the route length.
3. The limitation of the parking capacity at the metro stations is ignored, which means that the number of vehicles in one station is only dependent on travel demand. Travel delays caused by road congestion are also ignored.
4. Vehicles are encouraged to cycle to reduce no-load travel, which means that every vehicle is allowed to service a completely different route in another direction after it has finished its allotted route.

### 2.2 Integrated network representation

**Fig 2:**
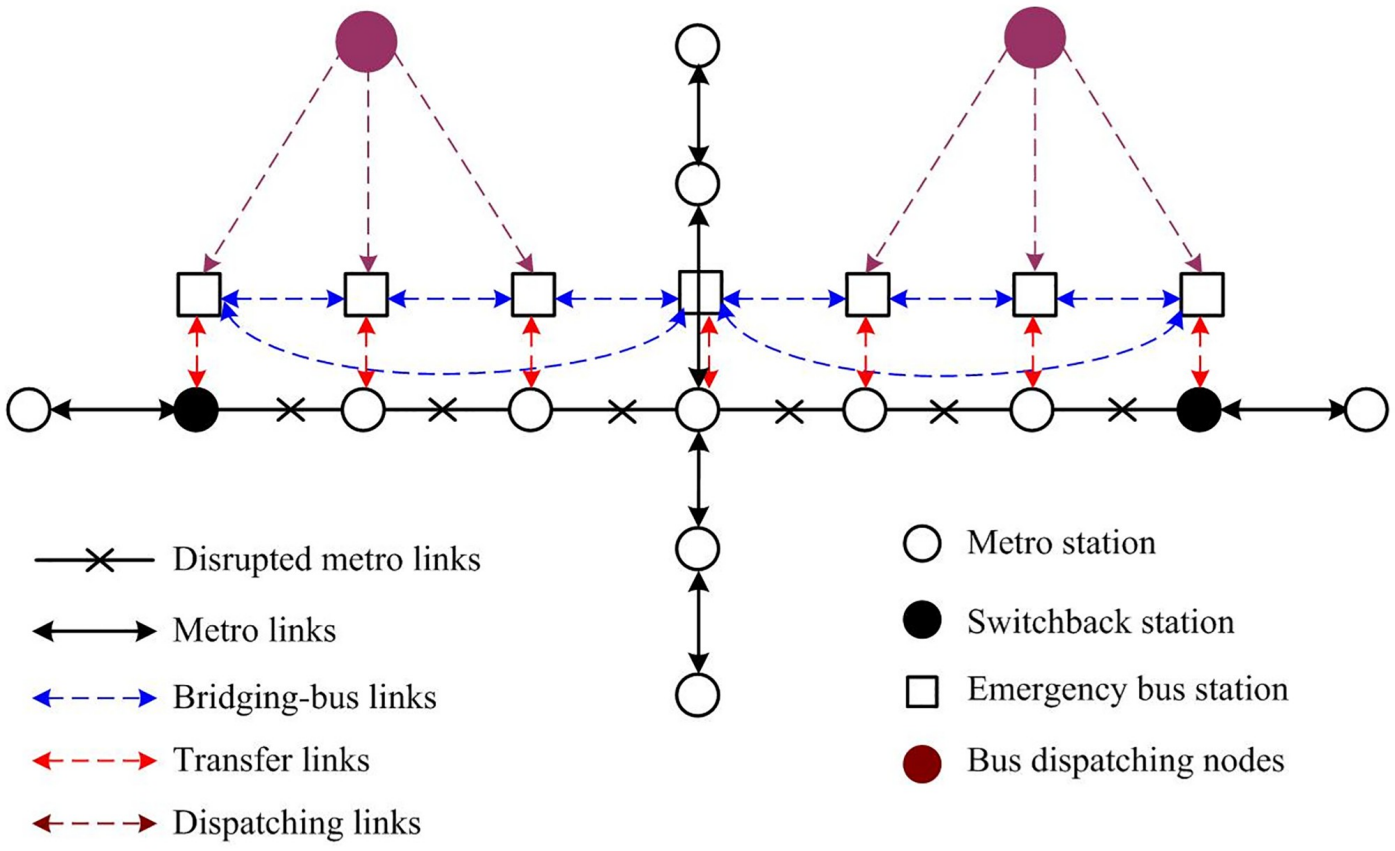
Representation of the integrated metro and bus network.

As shown in Fig 2, the integrated network can be represented by a directed graph *G(N*, *A)*, where *N* is the node set, including bus dispatching nodes, disrupted metro stations, and emergency bus stations, and *A* is the link set, including the links between metro stations, emergency bus stations, bus dispatching nodes, and disrupted metro stations. The virtual links represent the transfers between the metro and the buses.

### 2.3 Candidate bus bridging route generation

The K-shortest paths algorithm was applied to find an optimal set of bus bridging routes. Before introducing this method, the following notations are defined:

*R*: Set of candidate bus bridging routes, including the standard bus bridging route *r*_*1*_, as indicated by the red lines in Fig 1.
*К*: Set of candidate bus bridging links.
*D*: Set of emergency dispatching nodes.
*M*: Set of disrupted metro stations out of a total of *m* disrupted stations.
*OD*: Travel demand matrix.
*Q*: Total number of affected passengers during the disruption.
*Q*_*ij*_: Total number of affected passengers traveling from node *i* to node *j*, where *i*, *j* ∈ *M*.
$t_{d_{di}}$: Bus travel time from dispatching node *d* ∈ *D* to disrupted metro node *i* ∈ *M*.
$t_{r_{ij}}$: Average bus travel time along link (*i*, *j*).
*N*: Pre-established constant limiting the total number of candidate routes. The number of candidate routes is the same in each direction.
*T*_*0*_: Pre-established constant representing the threshold of the total travel time for one route, which includes both the travel time from emergency dispatching nodes to disrupted metro nodes and the travel time from one disrupted metro node to another.

The process to find good candidate bus bridging routes is as follows:

**Step 1:** Search the candidate links with the largest travel demand in one direction.

Sort the links (*i*, *j*) with the top 30% according to the value of *q*_*ij*_ and add them to the set of К.

**Step 2:** Search for candidate routes. Given the *N* and *T*_*0*_ limitations, the objective is to connect as many candidate links as possible to maximize the number of evacuated passengers, i.e.:

Objective:

$$Max\sum_{r=2}^{N/2}\sum_{(i,j)\in \kappa }^{}q_{ij}$$ (1)

Decision variables:

$\alpha_{ij}^{r}\in \left[0,1\right]$, if link (*i*, *j*) is covered by route *r*, then $\alpha_{ij}^{r}=1$; otherwise, $\alpha_{ij}^{r}=0$

*β*^*r*^ ∈ [0,1], if bus-bridging route *r* is employed, then *β*^*r*^ = 1; otherwise, *β*^*r*^ = 0

Restrictions:

$$\text{For}\;\text{route}\;r\;\left(r\geq 2\right),t_{d_{di}}+\sum_{i=1,i<j}^{m}t_{r_{ij}}\leq T_{0}$$ (2)

$$\text{For}\;\text{router}\;r\;\left(r\geq 2\right),Round(\frac{m}{3}+0.5,0)-1\leq \sum \alpha_{ij}^{r}\leq Round(\frac{m}{3}+0.5,0)$$ (3)

$$\text{For}\;\text{link}\left(i,j\right)\in К,\sum_{r=2}^{N/2}\alpha_{ij}^{r}\leq 2$$ (4)

$$\sum_{r=2}^{}\beta^{r}\leq \frac{N}{2}-1$$ (5)

Eq (1) represents the objective of the model, which consists of the maximization of the total number of passengers that can be evacuated by running the candidate bus bridging routes (except for the standard route). Eqs (2)–(5) are restrictions. Eq (2) indicates that the travel time of candidate route *r* (except for the standard route) should not exceed *T*_*0*_; it includes the dispatching time as well as the travel time among the disrupted stations. Eq (3) ensures that the upper and lower limits of the legs of the bus bridging route (except for the standard route). It can be proved that if the average stop time at one station is equal to 2 min, the travel time of candidate routes will save at least 2[*m*−*Round*(*m*/3+0.5, 0)−1] minutes more than the standard route because it can decrease the stop times. When *m* ≥ 9, the travel time of one candidate route can be reduced by at least 10 min. This is very helpful in decreasing the average delay. Eq (4) indicates that link (*i*, *j*) ∈ К shall not appear more than twice in all candidate routes, except for the standard route. Eq (5) represents the maximum number of candidate routes in one direction.

The solution of the model proceeds as follows:

1. Update the network graph according to the set of candidate bus bridging links. If a candidate link exists between any two nodes, a link is effectively added between them, and the adjacency and impedance matrices are updated.
2. Generate all possible combinations between the candidate route’s original and destination nodes subject to the restriction given by Eq (5). The route’s original and destination nodes selected in one direction can be connected to emergency nodes and should be as close as possible to the turn-back stations to facilitate the cycling operation.
3. Search for valid paths for one candidate route and calculate the number of passengers it can carry. Yen’s K-shortest paths algorithm is adopted to find valid paths based on the restrictions stated by Eqs (2)–(4) [27, 30]. If the shortest path does not satisfy these restrictions, the next k-shortest path is searched until all the restrictions are satisfied. Then, the number of OD demands that this path can meet is calculated. In this manner, we search and record candidate paths for a combination of all routes.
4. Choose the set of candidate routes that maximizes the OD demand.

**Step 3:** Repeat steps 1 and 2 to find candidate routes in another direction.

**Step 4:** Search for routes that can support the vehicle cycling operation. The decision regarding the vehicle cycle should be such that the destination of one direction and the origin of another are the same or connected by one edge. This aims to reduce the waiting time of passengers. It is worth mentioning that one vehicle is encouraged to cycle operations but the route must be fixed to avoid driver confusion.

### 2.4 Resilience-based bus bridging dispatching optimization model

Before optimization, the cross-sectional flow $Q_{ij}^{r}$ of route *r* should be obtained from traffic assignment. In this study, a logit model is adopted to calculate the probability of route *r*:

$$P_{w}^{r}=\frac{\text{exp}(-\varphi T_{w}^{r}/\bar{T_{w})}}{\sum \text{exp}(-\varphi T_{w}^{r}/\bar{T_{w})}},r\in R$$ (6)

$$T_{w}^{r}=t_{w}+\sum t_{r_{ij}}^{r}+\sum t_{s_{i}}^{r}$$ (7)

$$Q_{ij}^{r}=\sum_{w}q_{ij}P_{w}^{r}y_{ij}$$ (8)

Where:

$P_{w}^{r}$: Probability of selecting route *r* for OD pair *w*.
*φ*: Assignment parameter.
$T_{w}^{r}$: Travel time of route *r* for OD pair *w*.
$\bar{T_{w}}$: Average travel time for all candidate routes for OD pair *w*.
*R*: Set of candidate routes.
*t*_*w*_: Average waiting time for passengers, which equals the travel time of one vehicle from the emergency dispatching node to the passengers’ station.
$t_{r_{ij}}^{r}$: Bus riding time along link (*i*, *j*) on route *r*.
$t_{s_{i}}^{r}$: Bus stop time at disrupted node *i* on route *r*.
*y*_*ij*_: 0–1 variable. If link (*i*, *j*) belongs to route *r*, then *y*_*ij*_ = 1; otherwise, *y*_*ij*_ = 0.

Eq (6) is the probability that route *r* is selected for OD pair *w*. Eq (7) is the travel time of route *r* for OD pair *w*. Eq (8) is the cross-sectional flow of route *r*.

The resilience-based bus bridging and dispatching optimization is defined as follows:

Objective:

$$Max\int_{t_{0}}^{t_{\text{max}}}\frac{Q_{t}}{Q}dt\approx Max\sum \Delta t_{i}\frac{Q_{t_{i}}}{Q}$$ (9)

Decision variables:

$x_{di}^{r}$: Number of vehicles from emergency dispatching node *d* to disrupted metro node *i* on service route *r*.

$k_{b}^{r}$: Number of vehicles that require cycling on route *r*.

Restrictions:

$$\text{For}\;\text{emergency}\;\text{dispatching}\;\text{node}\;d,\sum_{r}\sum_{i}x_{di}^{r}\leq P_{d}$$ (10)

$$\text{For}\;\text{route}\;r,(x_{di}^{r}+k_{b}^{r})C_{b}\delta_{\text{max}}\geq \text{max}(Q_{ij}^{r})$$ (11)

$$\text{For}\;\text{any}\;\text{one}\;\text{vehicle},\text{max}[t_{d_{di}}^{}-t_{z}+\sum_{r}^{}\sum_{i=1}^{m}(t_{r_{ij}}^{r}+t_{s_{i}}^{r}+t_{z})]\leq t_{\text{max}}$$ (12)

$$\frac{\sum_{i,j\in M}\sum_{r\in R}q_{ij}P_{ij}^{r}(t_{wi}^{r}+t_{rij}^{r}+\sum t_{s}^{r}-t_{ij}^{m})+\psi \sum_{i,j\in M}\bar{q_{ij}}}{\sum_{i,j\in M}q_{ij}}\leq t_{d\text{max}}$$ (13)

Where:

*Q*_*t*_: Total number of passengers evacuated at time *t*.
Δ*t*_*i*_: 5-min time interval.
$Q_{t_{i}}$: Total number of passengers evacuated during Δ*t*_*i*_.
$x_{di}^{r}$: Number of vehicles sent from emergency dispatching node *d* to disrupted metro node *i* servicing route *r*.
*P*_*d*_: Number of available vehicles at emergency dispatching node *d*.
$Q_{ij}^{r}$: Cross-sectional flow of link (*i-j*) on route *r*.
$t_{w_{i}}^{r}$: Waiting time at disrupted node *i* on route *r*
$t_{r_{ij}}^{r}$: Bus riding time along link (*i*, *j*) on route *r*.
$t_{s_{i}}^{r}$: Bus stop time at disrupted node *i* on route *r*
*t*_*z*_: Average turn-back time of the vehicle.
*t*_*max*_: Pre-established maximum total evacuation time, which can be the same as the predicted metro operational disruption time.
$t_{ij}^{m}$: Metro average travel time along link (*i*, *j*) when there is no disruption.
*C*_*b*_: Bus capacity.
*δ*_*max*_: Maximum capacity rate of the buses.
*t*_*dmax*_: Pre-established maximum average delay for affected passengers.
*ψ*: Time penalty coefficient for passengers who cannot board a bus at the first opportunity.
$\bar{q_{ij}}$: Number of passengers of one OD pair who cannot board a bus at the first opportunity.

Eq (9) states the objective of the model, which consists of the maximization of the resilience index of the evacuated passengers. The integral is transformed into a cumulative summation to simplify the calculation. Eqs (10)–(13) are the model’s constraints. Eq (10) ensures that the total number of vehicles in emergency node *d* does not exceed *P*_*d*_, thus specifying a resource limitation. Eq (11) represents the bus capacity limitation; the capacity of bus bridging route *r* should be larger than its maximum cross-sectional flow. Eq (12) indicates that the maximum operation time of any vehicle shall not exceed *t*_*max*_, which includes the dispatching, riding, stop, and turn-back times. This study ignores the turn-back time in the last evacuation operation. Eq (13) guarantees that the average delay of all passengers does not exceed *t*_*dmax*_. Owing to the existing situation of some passengers who cannot board a bus at the first opportunity, travel time is weighted using a penalty coefficient *ψ*.

**Fig 3:**
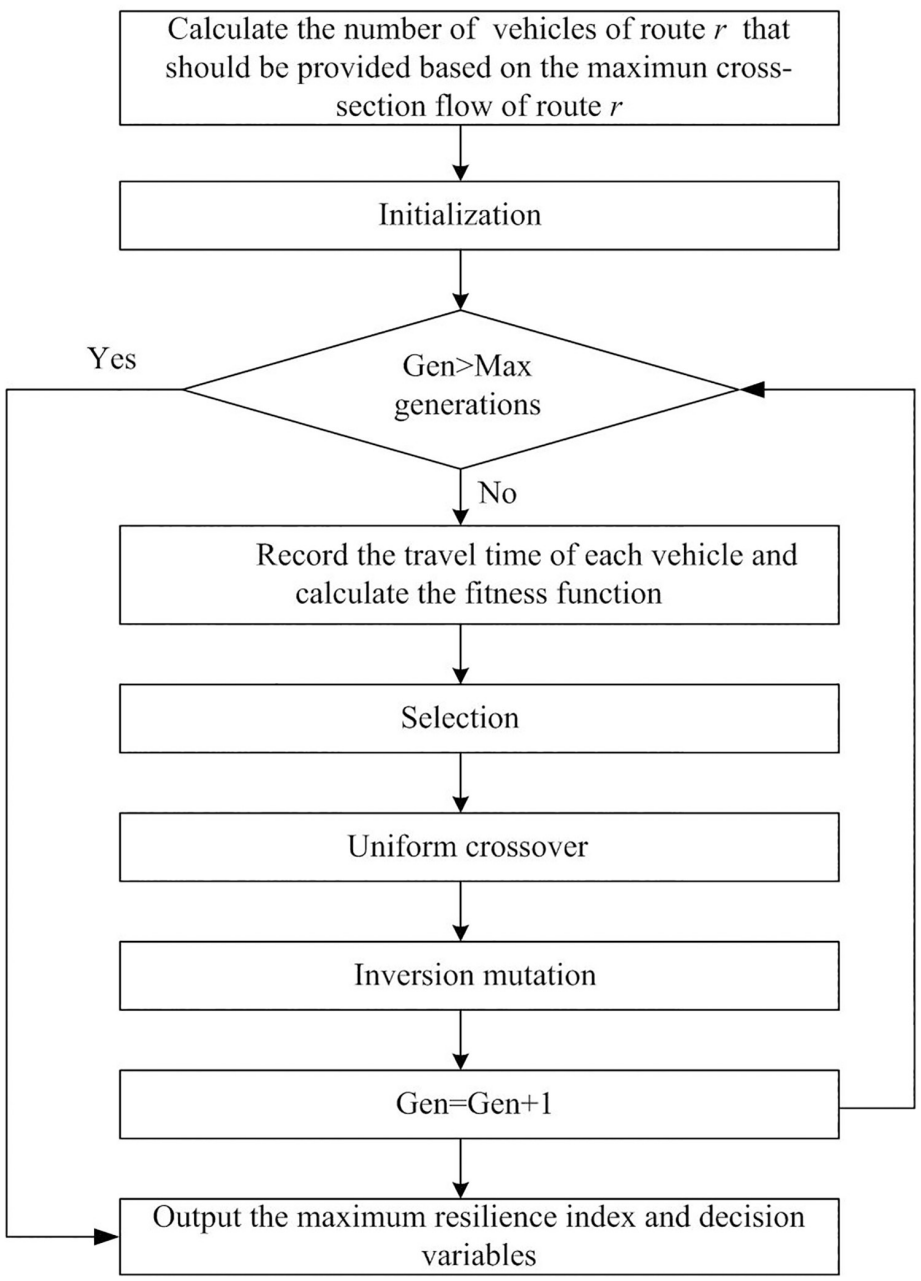
Flowchart of the model solution.

A flowchart of the solution is illustrated in Fig 3. A GA is adopted to solve the proposed model. The GA has four important parameters: the number of individuals in the population (population), maximum number of generations (max generations), crossover rate (*P*_*c*_), and mutation rate (*P*_*m*_). The crossover rate is used to determine the probability that two individuals crossover, whereas the mutation rate is used to determine the probability that any individual mutates. The fitness function is the resilience index of the evacuated passengers. The solution of the dispatching optimization model is dynamic because the resilience index must be updated for each set of decision variables. The GA terminates when the maximum number of iterations is reached or when the fitness function achieves no substantial improvement after a certain number of iterations [9].

### 3. Case study

#### 3.1 Scenario description

**Fig 4:**
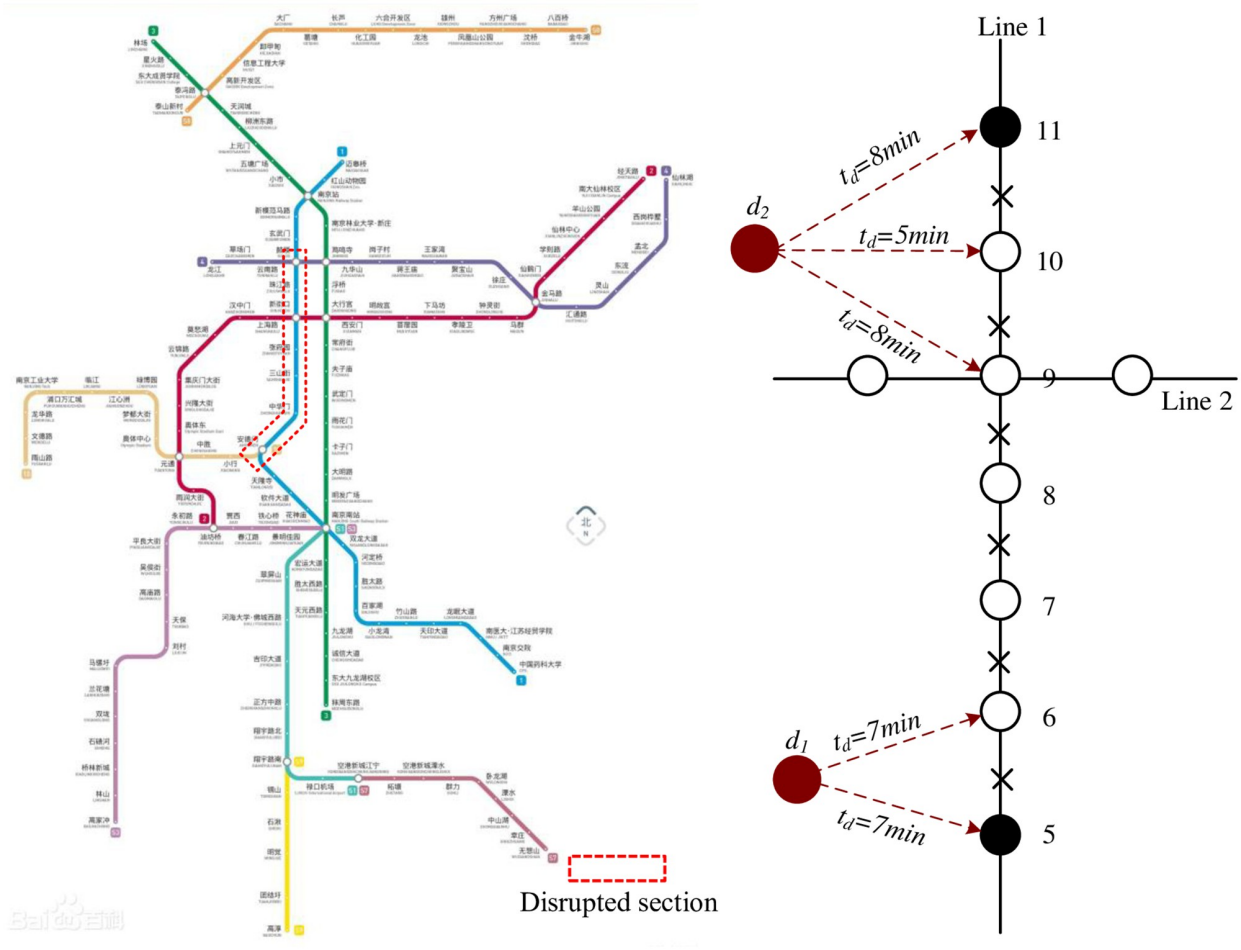
Schematic of the disrupted network considered in the case study.

To test the proposed model, a scenario consisting of the disruption of Line 1 of the Nanjing Metro, which includes 27 stations, is analyzed. Because of a signal failure, the section between stations Sanshanjie (Id:7) and Zhujianglu (Id:10) was disrupted at 10:00 am, with the nearest turn-back stations being Andemen (Id:5) and Gulou (Id:11). Operators predicted that the disruption could last for one hour, hence, the metro system had to operate in a short-turning mode. One metro line could operate between the terminal and Andeme (Id:5) and the other between the terminal and Gulou (Id:11), while the section between Andemen (Id:5) and Gulou (Id:11) was serviced by bus bridging. The buses came from adjacent depots or were retracted from existing bus routes. The bus bridging routes were parallel to the impacted metro line section. The available dispatching nodes and dispatching time to the affected stations are shown in Fig 4.

Because the disruption could last for one hour, the maximum evacuation time was set to 60 min. During metro disruptions, commuters’ waiting time is usually quite long. Thus, the maximum average delay was set to 30 min. Other model parameters are listed in Table 1.

**Table 1:** The parameters of model.

Symbol	Explanation	Value	Symbol	Explanation	Value
***φ***	Assignment parameter	8.9	*δ*_*max*_	Bus maximum capacity rate	1.2
*t*_*z*_	Bus turn back time	2*min*	*t*_*s*_	Bus average stop time	1*min*
*C*_*b*_	Bus capacity	75	*ψ*	Time penalty coefficient	50
*t*_*dmax*_	Upper of average delay	30*min*	*t*_*max*_	Upper of total evacuation time	60*min*

We assume that the evacuation demand is known and fixed, and can be derived from the historical records of the Automatic Fare Collection (AFC) system, which can collect smart card data, including card ID, time, boarding station, and alighting station. The OD demand obtained by analyzing the historical data and corresponding to the affected passengers, including those stranded at the start of the disruption and those arriving during the succeeding 15 min, is presented in Table 2. The riding times of the metro and buses for the different OD pairs are listed in Table 3.

**Table 2:** The affected passengers’ OD demand.

Id	5	6	7	8	9	10	11	Sum
5	0	28	78	66	167	102	96	536
6	32	0	78	74	242	155	137	718
7	90	67	0	23	230	123	138	671
8	69	63	28	0	95	72	91	418
9	185	150	195	87	0	166	199	982
10	112	124	142	63	169	0	59	668
11	99	119	156	80	286	54	0	793
Sum	587	550	677	393	1188	670	721	4787

**Table 3:** The riding time of metro/bus (min).

Metro/Bus	5	6	7	8	9	10	11
5	-	4/8	7/14	8/15	10/21	12/25	15/31
6	4/8	-	3/6	5/10	6/13	8/17	10/21
7	7/14	3/6	-	2/4	3/6	5/11	7/15
8	8/15	5/10	2/4	-	2/4	4/9	5/12
9	10/21	6/13	3/6	2/4	-	2/5	3/7
10	12/25	8/17	5/11	4/9	2/5	-	2/4
11	15/31	10/21	7/15	5/12	3/7	2/4	-

### 3.2 Candidate bus bridging routes

**Fig 5:**
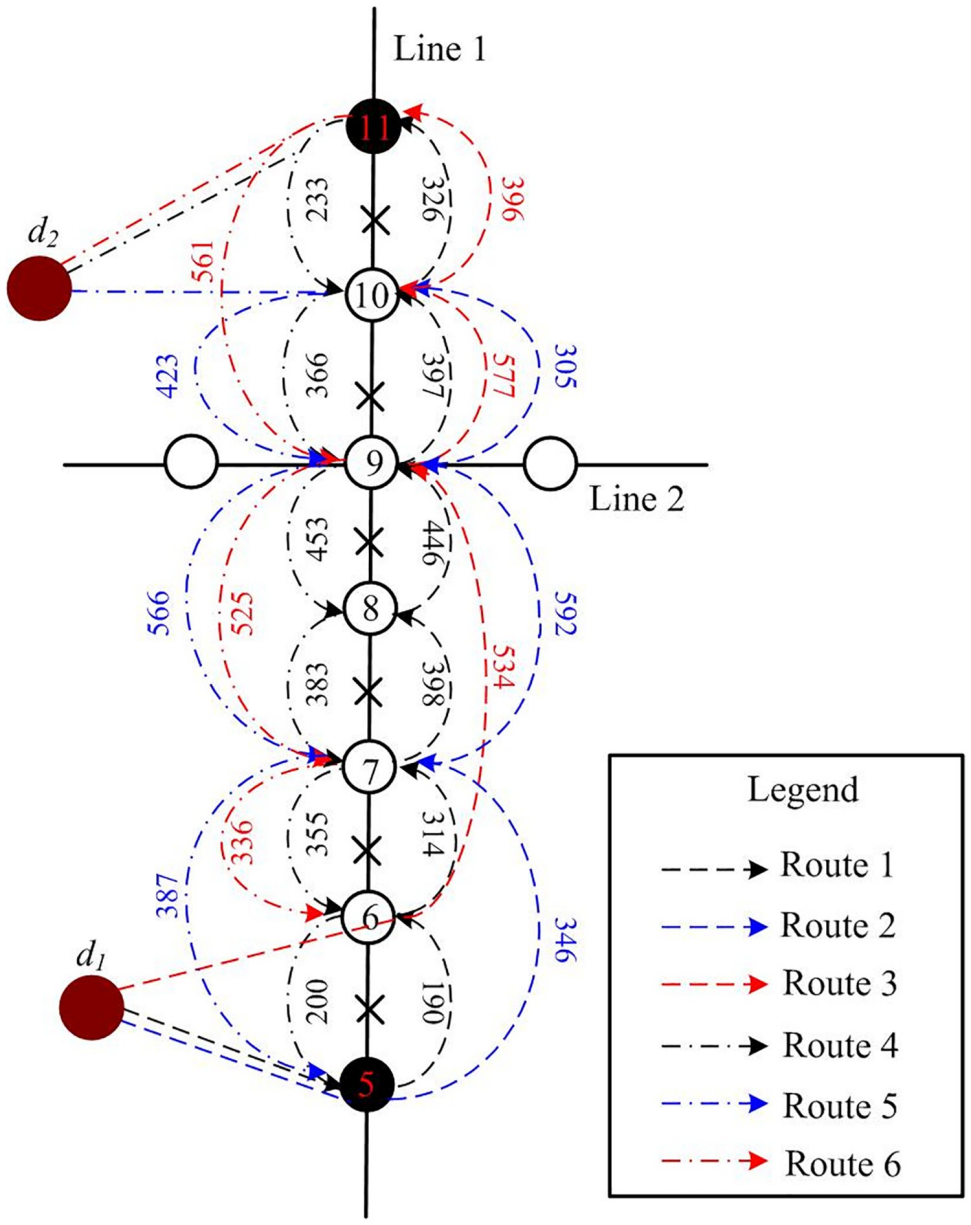
The candidate bridging-bus routes in scenario.

By sorting the affected passengers’ demand in Table 2, the top 30% demand corresponds to links 6–9,7–9,9–11,5–9,9–10,6–10 (Id: i<j) and 11–9,9–7,9–5,10–9,11–7,9–6 (Id: i>j). The candidate bus bridging routes and travel times when *N* = 6 and *T*_*0*_ = 35 min are shown in Fig 5 and Table 4. As mentioned before, cycle operation is allowed for all buses; therefore, routes 1 and 4, 2 and 5, and 3 and 6 can be used as cycling pairs.

**Table 4:** The links and travel time of candidate bridging-bus routes in scenario.

Name	Links	Time(min)	Name	Links	Time(min)
Route 1	*d*_*1*_ *-5-6-7-8-9-10-11*	38	Route 4	*d*_*2*_ *-11-10-9-8-7-6-5*	39
Route 2	*d*_*1*_ *-5-7-9-10*	32	Route 5	*d*_*2*_ *-10-9-7-5*	30
Route 3	*d*_*1*_ *-6-9-10-11*	29	Route 6	*d*_*2*_ *-11-9-7-6*	27

### 3.3 Optimal scheme for different bus resources

Using the logit model described in Section 2.4, the cross-sectional flow of each route is shown in Fig 5. To illustrate the sensitivity of resources, this study compared the optimal dispatching schemes with different resources, as shown in Table 5. Because of the limitation of available vehicles, cycle operations need to be adopted in conditions such as *C*: *P*_*1*_ = 15 and *P*_*2*_ *= 16*. Routes 2, 3, 5, and 6 require two buses to cycle. When comparing *C* and *B*, the number of available vehicles decreased to 8, whereas the total evacuation time, average waiting time, and average delay increased. In particular, the total evacuation time increased by 24.4%, but the average delay increased by only 3.05 min.

**Table 5:** The sensitive analysis of the number of available bus vehicles.

		*A*:*P*_*1*_ = *P*_*2*_ *= 22*	*B*:*P*_*1*_ = 19, *P*_*2*_ *= 20*	*C*:*P*_*1*_ = 15, *P*_*2*_ *= 16*	*D*:*P*_*1*_ = 15, *P*_*2*_ *= 16*
The number of vehicles	Route 1	6	5	5	5
	Route 2	8	7	5+2 of Route 5	5+2 of Route 2
	Route 3	8	7	5+2 of Route 6	5+2 of Route 3
	Route 4	7	6	6	6
	Route 5	7	7	5+2 of Route 2	5+2 of Route 5
	Route 6	8	7	5+2 of Route 3	5+2 of Route 6
The number of vehicles that need cycling operate		0	0	8	0
The total evacuation time		45min	45min	56min	95min
The resilience index		0.530	0.530	0.499	0.481
The average waiting time		19.57min	19.57min	22.62min	22.89 min
The average delay		26.26min	26.26min	29.31min	29.57 min
The maximum utilization rate of bus capacity		1.078	1.189	1.2	1.2
The minimum utilization rate of bus capacity		0.381	0.444	0	0
The average utilization rate of bus capacity		0.862	0.895	0.885	0.885
The percent of passengers who need second waiting		0	0	4.69%	7.99%

The utilization rate of the bus capacity of *C* is interesting, reaching the upper and lower limits. For example, in Route 2, the difference in cross-sectional flow is significant, so the utilization rate of the bus capacity reaches one in the first round of operation. However, when one vehicle cycle operates on Route 2, link 5–7 does not have travel demand, so the utilization rate of bus capacity equals zero.

To demonstrate the advantage of the cycling operation, another dispatching strategy was adopted in Case *D*. The strategy consists of servicing fixed lines with fixed vehicles, which means that if passengers of one link in one route are not evacuated in the first round, the vehicle in this route must return to evacuate them. For example, 5 vehicles serve Route 5, but passengers of link 9–7 are stranded because of limited vehicles, and two vehicles must return to evacuate them from station 5 to station 9. However, if a vehicle cycling strategy is adopted, passengers will be evacuated by vehicles from Route 2. By adopting the former strategy, the waiting time of passengers of link 9–7 increases by 16 min. Thus, the total evacuation time, average waiting time, and average delay of *D* increase compared with those of *C*. Although the average waiting time and average delay do not significantly change, some indicators even exceed the pre-established restrictions, such as the total evacuation time, which may exceed 90 min. This is because 7.99% of passengers need a second waiting period, extending their waiting time to over 60 min.

To illustrate the sensitivity of resources, this study also compared a case with adequate resources. In Scheme *A*, compared with *B*, the number of available vehicles increases by 5, but not the utilization rate of bus capacity; other indicators remain the same. Considering only the utilization rate of the resources, the performance of *B* is better than that of *A*. Moreover, under the limitation of *B*, the resilience index is the largest, and all affected passengers can be evacuated at one time. By analyzing the different resource limitations, we can also conclude that when bus resources increase up to a certain value, the indicators do not significantly change, and too much input may waste resources. However, with limited resources, the cycling strategy can effectively resolve the problem of a second waiting period and optimize the vehicles’ travel path, as well as significantly decrease the travel delay.

**Fig 6:**
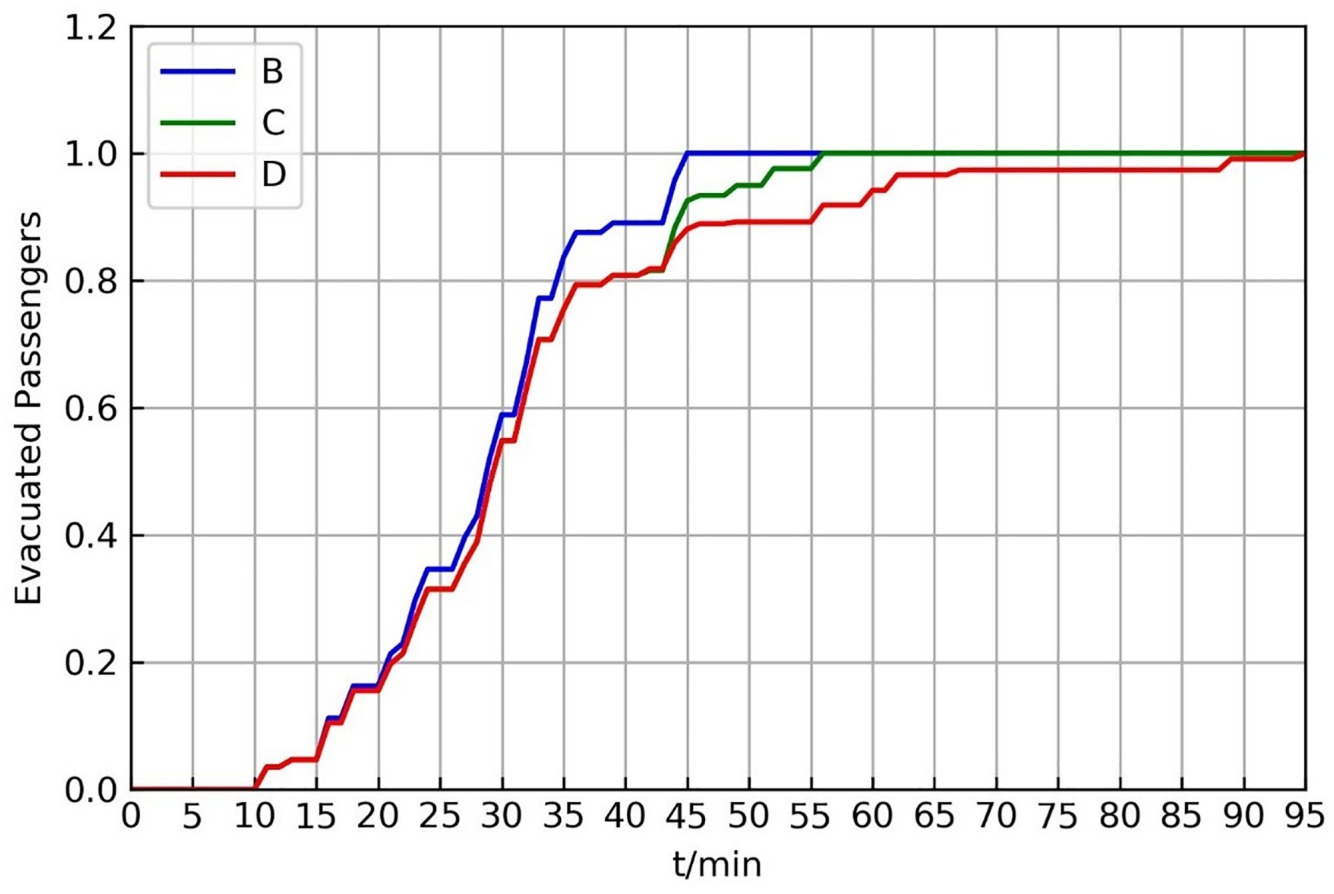
Optimal schemes for different resource limitations.

Fig 6 illustrates the resilience curves for the different schemes. The resilience curve can provide more details about the evacuation process. From 0 to 20 min, the difference between schemes was very small. From 20 to 42.5 minutes, the resilience curves of schemes *C* and *D* were the same. This is because the vehicle resource allocation was also the same during this time. After 42.5 min, when a cycling operation strategy was adopted in scheme *C*, vehicles could reach the affected stations as soon as possible, and the stranded passengers could be efficiently evacuated. The total evacuation time of scheme *C* shortens by approximately 30 min compared to that of scheme *D*. Thus, the resilience index of scheme *C* improved by 3.74% compared to that of scheme *D*. Scheme *B* has more adequate vehicle resources than the other schemes; as a result, the evacuated passenger resilience curve recovers faster. Passengers can be evacuated quickly between 10 and 36 min, but the evacuation speed decreases between 36 and 43 min. However, the total evacuation time of scheme *B* shortens by 10 min compared to that of *C*.

**Fig 7:**
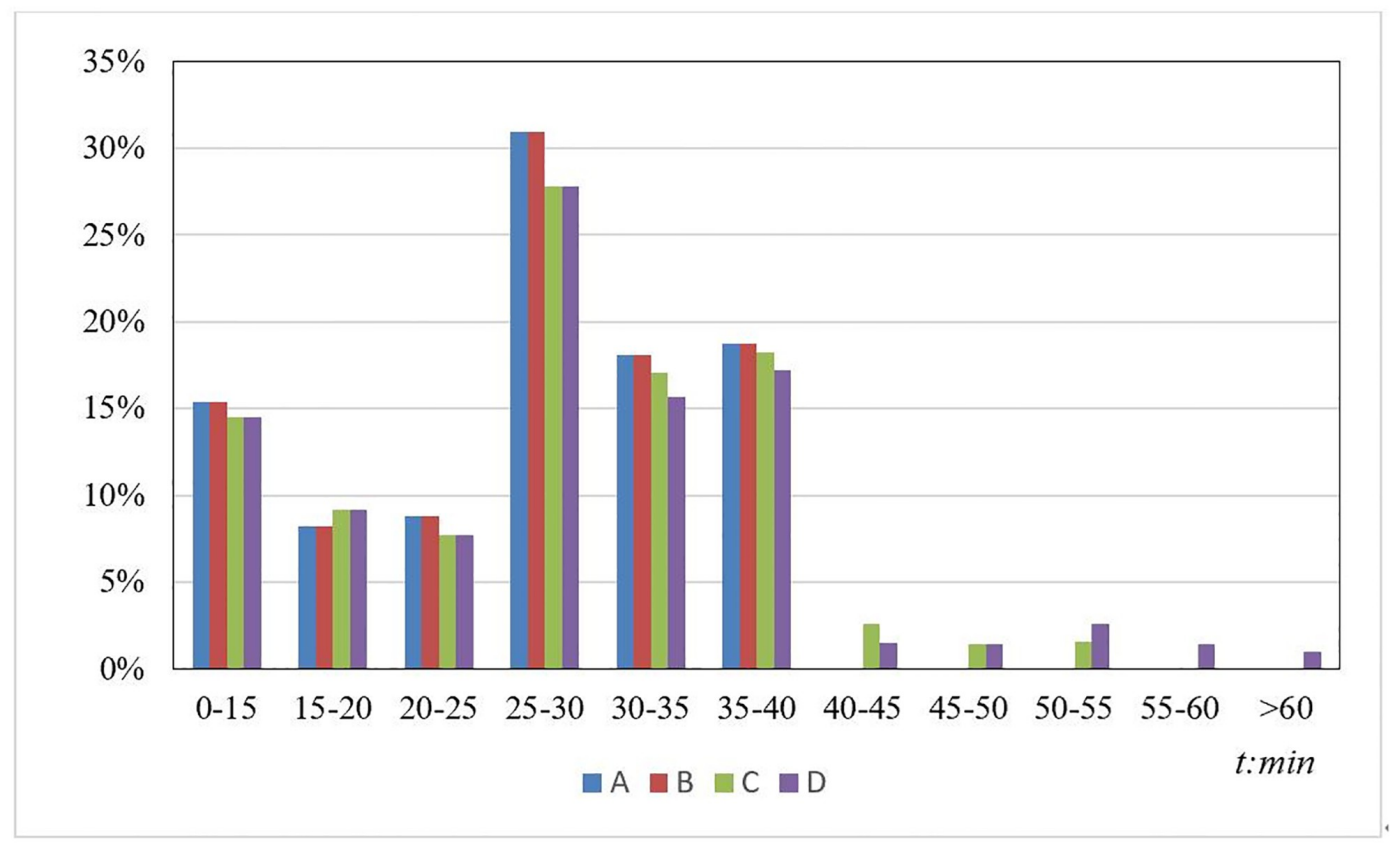
Travel delays for different resource limitations.

Fig 7 shows the travel delays for different resource limitations. Although the difference in average travel delay is very small, 5.55% and 7.94% of the passengers experience delays of over 40 min in schemes *C* and *D*, respectively, with the longest delay exceeding 60 min, which is unacceptable to passengers. The main reason for travel delay was the average waiting time. Thus, a timely bus bridging response and a low dispatching time from emergency nodes to disrupted stations significantly improve the efficiency of the evacuation process. Furthermore, a reasonable route design helps decrease the average delay for all passengers.

### 3.4 Algorithm performance

**Fig 8:**
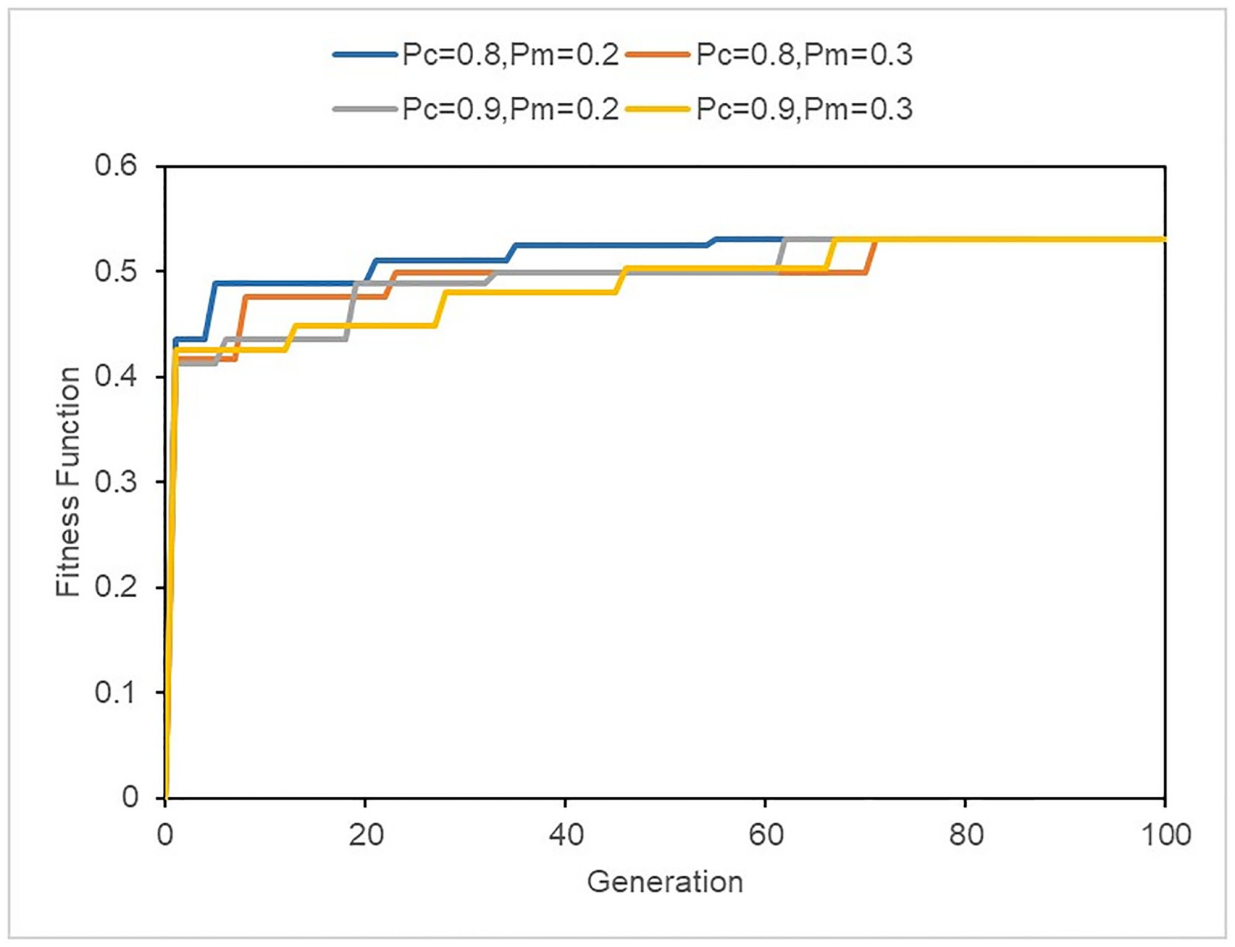
Convergence of the GA under different parameters.

We solved the bus bridging vehicle dispatching model using a GA. The computational time was approximately 4 min on a PC with a 3.7 GHz Intel Core CPU and a 4.0 GB memory. The convergence of the GA under different parameters based on resource limitation *C* is shown in Fig 8 (population = 100, maximum generations = 100). The best performance was achieved by combining a crossover rate of *P*_*c*_ = 0.8 with a mutation rate of *P*_*m*_ = 0.2, which arrives at the same solution before the 58th generation, indicating a fast convergence and good stability.

### 3.5 Sensitivity analysis of evacuation demand

In practice, the evacuation demand fluctuates because the travel behavior of passengers is difficult to describe. This section analyzes the sensitivity of the model to the fluctuating evacuation demand with fixed vehicle resources (*P*_*1*_ = 17, *P*_*2*_ *= 18*). The basic evacuation demand was quantified using an OD matrix (Table 2). Assuming that the fluctuating evacuation demand is proportional to the basic demand, Table 6 lists the optimal dispatching scheme indicators for different evacuation demands.

**Table 6:** The sensitivity analysis of fluctuating evacuation demand.

		85%Q	90%Q	Q	105%Q	110%Q
Number of dispatched vehicles	Route 1	5	5	5	5+1 of Route 4	5+1 of Route 4
	Route 2	6	6	6+1 of Route 5	6+1 of Route 5	6+1 of Route 5
	Route 3	6	6+1 of Route 6	6+1 of Route 6	6+1 of Route 6	6+2 of Route 6
	Route 4	6	6	6	6	6+1 of Route 1
	Route 5	6	6	6+1 of Route 2	6+1 of Route 2	6+1 of Route 2
	Route 6	6	6+1 of Route 3	6+1 of Route 3	6+1 of Route 3	6+1 of Route 3
Number of vehicles that need cycling operate		0	2	4	5	7
Total evacuation time		45min	51min	55min	58min	63min
Resilience index		0.539	0.513	0.502	0.482	0.465
Average waiting time		16.01min	19.21min	21.64min	23.09min	25.12min
Average delay		22.21min	27.35min	28.28min	29.88min	31.85min
The maximum utilization rate of bus capacity		0.96	1.20	1.20	1.20	1.20
The minimum utilization rate of bus capacity		0.85	0.25	0	0.13	0.21
The average utilization rate of bus capacity		0.912	0.893	0.883	0.887	0.891
The percent of passengers who need second waiting		0%	1.22%	3.58%	4.93%	6.26%

Existing vehicle resources can fully evacuate 85% of the affected passengers. In this case, passengers do not need to wait a second time. The delay of passengers is the smallest and the average utilization rate of vehicle capacity is the highest. When the evacuation demand fluctuates between -10% and +5%, the cycle operation strategy needs to be adopted. When the evacuation demand exceeded 5%, the average delay of passengers and the total evacuation time surpassed the threshold value. In this case, existing vehicles cannot meet the evacuation demand and other resources need to be urgently allocated.

**Fig 9:**
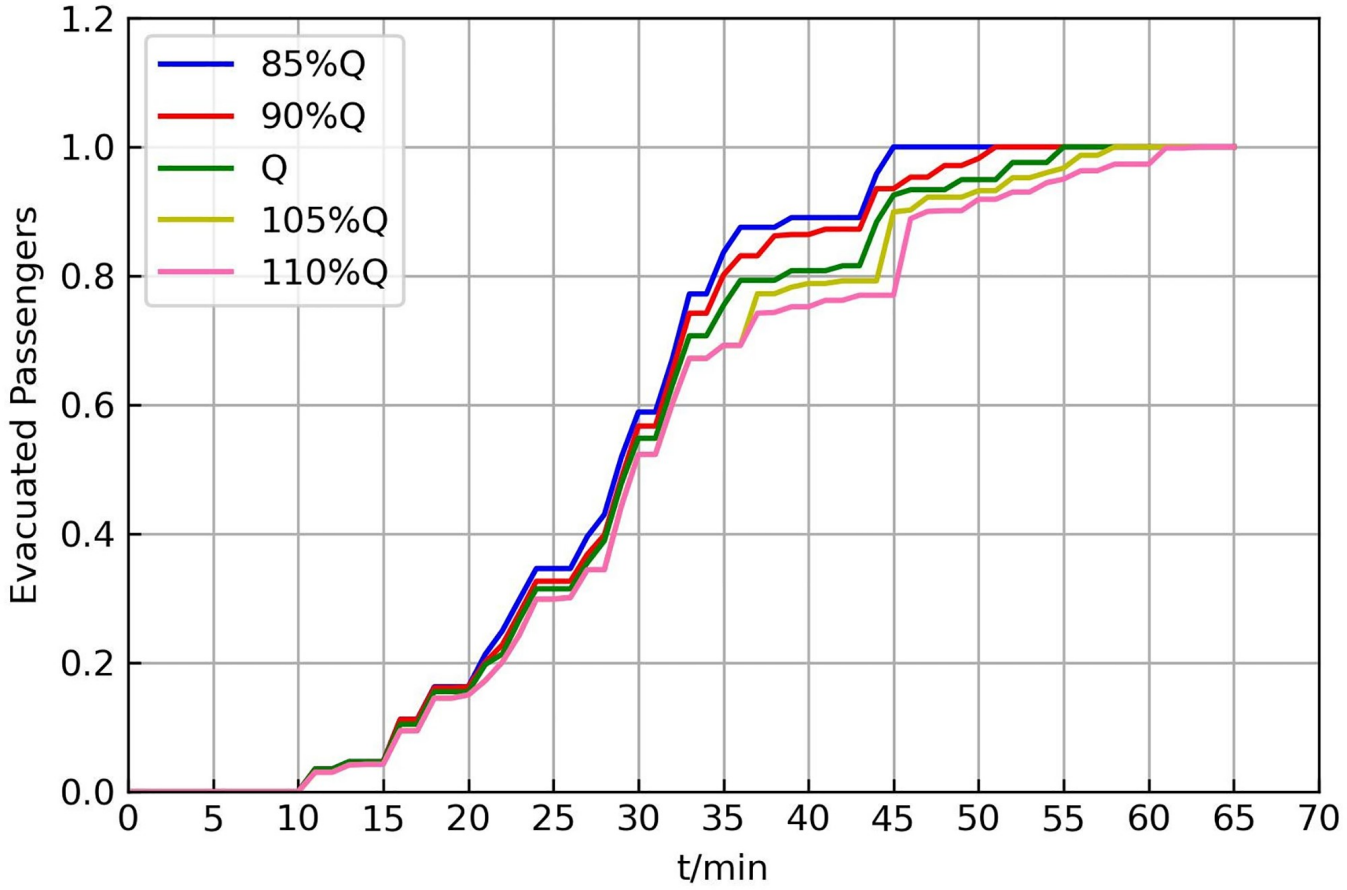
Resilience curves for different evacuation demands.

Fig 9 illustrates the evacuated passenger resilience curve for different demands. The difference was very small before 33 min. Owing to the limitation of vehicle resources, as demand increases, more passengers need a second waiting, which gradually decreases the evacuation efficiency and progressively increases the total evacuation time.

## 4. Discussion

To verify the advantages of the proposed model, it was further compared with the work of Li et al. [9], who proposed an emergency dispatching optimization model to minimize the total evacuation time of stranded passengers with a fixed headway. Table 7 lists the corresponding dispatching schemes and main indicators using the same resource limitation (*P*_*1*_ = 17, *P*_*2*_ *= 18*) and different optimization objectives. It is evident that the proposed model can effectively reduce the total evacuation time because of the cycling dispatching strategy. In addition, the proposed model assumes that the headway equals zero; therefore, the average delay of passengers reduces by 3.51%. This assumption is suitable for unexpected and short-term emergency evacuations. Moreover, the proposed model aims to maximize the resilience index of the affected passengers. The resilience index not only reflects the total evacuation time but also focuses on the evacuation process.

**Table 7:** The comparison of different optimization models.

		Maximize total evacuation time	Maximize affected passengers’ resilience index
Number of dispatched vehicles	Route 1	5	5
	Route 2	6+1 of Route 2	6+1 of Route 5
	Route 3	6+1 of Route 3	6+1 of Route 6
	Route 4	6	6
	Route 5	6+1 of Route 5	6+1 of Route 2
	Route 6	6+1 of Route 6	6+1 of Route 3
Number of vehicles that need cycling operate		0	4
Total evacuation time		62min	55min
Resilience index		-	0.502
Average waiting time		23.01min	21.64min
Average delay		29.31min	28.28min
The average utilization rate of bus capacity		0.876	0.883

The proposed model can provide a near-optimal emergency bus bridging and dispatching plan based on fixed passenger demand and resource limitations. Especially in the case of very limited resources, a vehicle cycling strategy can play an important role in reducing no-load travel and, to a great extent, avoiding a second waiting time for some passengers. Realistically, some passengers may not be able to stand waiting for long periods and choose other means of transportation, which may lead to a loss of demand. Even considering this possibility, a vehicle cycling strategy can minimize unnecessary driving. Ultimately, all vehicles need to return to the emergency dispatching nodes, as they can evacuate passengers on their way back. In addition, because the metro agency can predict the second waiting period for passenger distribution using the route choice model, they can ask for extra help from other transit agencies to lighten the pressure by employing of additional vehicles, such as tourist and customized shuttle buses.

If vehicle resources are adequate, the proposed model can also provide a near-optimal dispatching plan to ensure that all passengers are evacuated at the first opportunity and to maximize the resilience index. The resilience index of evacuated passengers not only reflects the adequacy of the total recovery time but also illustrates the efficiency of the recovery process. This can prove very useful for transit agencies in implementing rescue operations.

## 5. Conclusions and future work

The innovation of the proposed model lies in the combination of resilience evaluation and dispatching optimization to maximize the resilience index of evacuated passengers from the viewpoint of the entire evacuation process. Similar to previous research, this model considers the total evacuation time, average travel delay, and limitations of the vehicle resources, but unlike some studies that focus on the optimization of the vehicles’ headway, this study adopted a cycling strategy to reduce no-load travel, allowing each vehicle to run on different routes in different directions. The conclusions drawn from the case study are as follows:

1. The average waiting time is the main reason for travel delays, particularly for short-distance travel. In practice, timely bus bridging response, increasing emergency dispatching nodes to decrease dispatching time, and reasonable bus bridging route designs can reduce the travel delay caused by operational disruptions.
2. The number of available bus bridging vehicles directly determines evacuation efficiency. When there are very few available vehicles, travel delays are too long and many passengers need a second waiting time. However, with the increase in available vehicles, particularly when the number of vehicles exceeds a boundary, the improvement in the indicator is not significant.
3. The cycling strategy is beneficial for reducing the average travel delay and improving evacuation efficiency under limited vehicles, and advanced management technology can make it more applicable. In particular, in the case of very limited resources, the vehicle cycling strategy provides significant advantages over fixed vehicles servicing fixed lines.

The results and methods of this study can further aid decision makers in evaluating dispatching plans and their impact on evacuation efficiency, as well as provide effective dispatching strategies and tools to maximize the resilience index of evacuated passengers. This will contribute to enhancing public transit resilience and reliability, improving resource utilization and increasing evacuation efficiency.

This study assumed that the travel demand is fixed; however, in practice, some passengers who do not informed the disruptions may enter the metro system. Moreover, some affected passengers may choose another transportation mode to reach their destination. These factors can result in travel demand fluctuations. Therefore, in future work, travel demand during metro operational disruptions should be carefully analyzed. Optimizing dispatching based on the fluctuations of travel demand and a more precise route choice model can improve the efficiency and reliability of emergency responses.

## Supporting information

S1 Fig Schematic of the disrupted network considered in the case study. (TIF)

S1 Table The affected passengers’ OD demand. (PDF)
