# Supplementary figures and images for: Resilience-based optimization model for emergency bus bridging and dispatching in response to metro operational disruptions
Source: PLoS One. 2023 Mar 29;18(3):e0277577. doi: 10.1371/journal.pone.0277577 (PMC10057812; doi:10.1371/journal.pone.0277577)

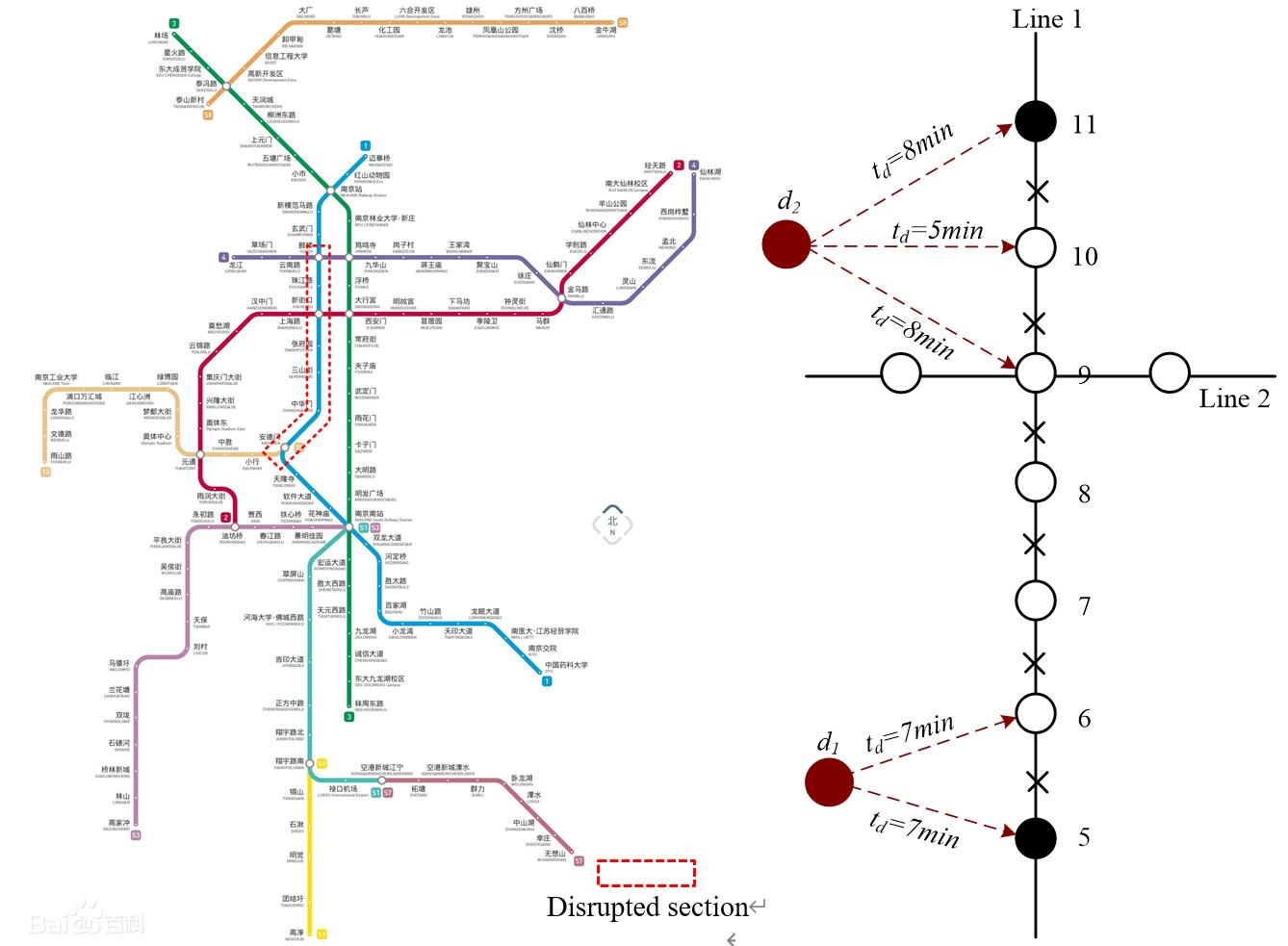

Supplement: S1 Fig — (TIF) [file pone.0277577.s001.tif]
