# Supplementary material for: Resilience-based optimization model for emergency bus bridging and dispatching in response to metro operational disruptions
Source: PLoS One. 2023 Mar 29;18(3):e0277577. doi: 10.1371/journal.pone.0277577 (PMC10057812; doi:10.1371/journal.pone.0277577)
Supplement: S1 Table — (PDF) [file pone.0277577.s002.pdf]

S1 Table. The affected passengers' OD demand

Id	5	6	7	8	9	10	11	Sum
5	0	28	78	66	167	102	96	536
6	32	0	78	74	242	155	137	718
7	90	67	0	23	230	123	138	671
8	69	63	28	0	95	72	91	418
9	185	150	195	87	0	166	199	982
10	112	124	142	63	169	0	59	668
11	99	119	156	80	286	54	0	793
Sum	587	550	677	393	1188	670	721	4787
